# Evaluation of crossbreeding strategies for improved adaptation and productivity in African smallholder cattle farms

**DOI:** 10.1186/s12711-025-00952-8

**Published:** 2025-02-20

**Authors:** Sèyi Fridaïus Ulrich Vanvanhossou, Tong Yin, Gregor Gorjanc, Sven König

**Affiliations:** 1 Institute of Animal Breeding and Genetics, Justus-Liebig-University Gießen, 35390 Gießen, Germany; 2 The Roslin Institute and Royal (Dick) School of Veterinary Studies, University of Edinburgh, Edinburgh, UK

## Abstract

**Background:**

Crossbreeding is successfully implemented worldwide to improve animal productivity and adaptability. However, recurrent failures of crossbreeding programmes in African countries imply the need to design effective strategies for the predominant smallholder production systems.

**Methods:**

A comprehensive simulation procedure mimicked body weight (BW_L_) and tick count (TC_L_) incidence in a local taurine cattle breed and in an exotic indicine beef cattle breed (BW_E_ and TC_E_, respectively). The two breeds were crossed to produce F1 and rotational animals. Additionally, synthetic breeds were created by applying four schemes defined as farm bull (FB), intra-village bull (IVB), exchanged-village bull (EVB), and population-wide bull (PWB) scheme. These schemes reflect different strategies to select and allocate bulls to smallholder farms. The different crosses were compared with the local breed over 20 generations by varying the genetic correlation between the traits (${r}_{g}$ = − 0.4, 0, 0.4), genotype-by-environment effects (GxE) between local and exotic environment (${r}_{g\times e}$ = 0.4, 0.6, 0.8), and the relative emphasis of TC_L_ compared to BW_L_ in a selection index (SI_TCL10%, SI_TCL30%, SI_TCL50%).

**Results:**

Regardless of ${r}_{g}$ and ${r}_{g\times e}$, EVB achieved the highest phenotypic and genetic gains for BW_L_ and TC_L_ over the 20 generations with SI_TCL50%. However, EVB displayed lower phenotypic means than F1 crosses in the first seven generations due to the loss of heterosis. Additive genetic variances were generally larger in synthetic crosses than in F1 and local animals, explaining the larger responses to selection. In addition, the EVB was the most effective strategy to stabilize inbreeding and retain heterosis in the advanced generations of synthetic animals. Low emphasis on TC_L_ (SI_TCL30%, SI_TCL10%) resulted in negative phenotypic gain for TC_L_ in synthetic animals when r_g_ = − 0.4. In contrast to F1 and rotational crosses, GxE effects did not affect phenotypic gain in synthetic crosses.

**Conclusions:**

The study demonstrates opportunities for long-term genetic improvement of adaptive and productive performances in smallholder cattle farms using synthetic breeding. Extensive exchange of semen between villages or regions controls inbreeding and additionally contributes to increasing genetic gain. Furthermore, the definition of a suitable selection index prevents antagonistic selection responses caused by negative correlations between traits and GxE effects.

**Supplementary Information:**

The online version contains supplementary material available at 10.1186/s12711-025-00952-8.

## Background

Innovative strategies are imperative for the implementation of successful crossbreeding programs in African countries, due to the huge demand of the rapidly growing African population on animal products. In the past, there have been numerous initiatives to crossbreed local and exotic breeds in African countries, but most of them have failed to increase meat and milk production [1, 2]. For instance, Ankole × Friesian and Ankole × Jersey crosses in Rwanda respectively produced only 4.01 L and 3.36 L of milk per day [3]. Similarly, body weight performances of crossbred animals are largely below those observed in other tropical regions (e.g., Latin America) [4, 5]. These results contrast with the worldwide success of crossbreeding and indicate the necessity to develop suitable breeding strategies [6, 7].

There is a lack of scientific studies addressing the question how crossbreeding can be effectively designed in an African production system context [1]. In Africa, livestock production environments are highly diversified with predominance of extensive smallholder production systems. The small size of the herds and the high genetic variability between them challenge reliable animal evaluation and selection [8–10]. In addition, the lack of infrastructures limits the application of genomic selection [11, 12]. Climatic conditions and increasing population density reduce the availability of forage, while most livestock herds rely on communal grazing [13, 14]. In addition, fluctuating temperatures and humidities increase disease pressures and heat stress on animals [15]. In such a variable stressful production context, smallholders favour robust animals with stable productivity [16]. Crossbreeding programs are rarely designed directly for and within smallholder production herds. One common crossbreeding strategy includes structured on-station approaches to generate improved breeding stock, which are later distributed to smallholder farmers. Another strategy is to generate crossbred animals directly on farm via insemination. Unfortunately, both of these strategies often fail to meet the preferences of farmers and are less adapted to smallholder production environments [5]. Reasons for these failures are often unclear and highly complex, partially due to the challenge of genotype-by-environment (GxE) interactions.

According to Mueller et al. [17], structured crossbreeding is not feasible in African smallholder livestock breeding contexts. The authors proposed the sporadic introduction of exotic genes as a pragmatic solution. However, non-systematic utilisation of exotic breeds implies non-predictable effects in ongoing generations. In addition, the non-systematic introduction of exotic breeds implies the substitution of locally adapted indigenous purebreds, threatening smallholder farming income, which is already challenged by the harsh environment. Alternatively, a pragmatic strategy for crossbreeding could be to customize terminal, rotational, or synthetic breeding strategies to the smallholder farming contexts [1, 2]. The terminal strategy involves selecting only local and exotic breeds and crossing them to produce terminal F1 progeny. In rotational strategy, the crossbred progenies are selected and crossed with sires from the opposite breed compared to previous crossing. In the synthetic breeding strategy, local and exotic breeds are first crossed, and then only crossbred populations are selected to produce future generations [18]. Terminal (F1) and rotational crossing schemes maximise heterosis, especially implying benefits for the improvement of low heritable or adaptive traits. Nevertheless, these strategies require supply of genetic materials from both local and exotic breeds in the long term. In addition, F1 animals from the crossing of local African breeds with exotic breeds are particularly affected by GxE effects, because the proportion of exotic genes in F1 animals is often too high for many smallholder settings. Such animals are frequently reported to have decreased adaptive performances (e.g., resistance to diseases and heat stress) compared to local animals, which is consequently often accompanied with a decline in productivity (meat, milk) [5]. The creation of a synthetic breed could support the production of crossbred animals which are adapted to local conditions, while benefiting from high productive performances of exotic breeds [1]. Synthetic populations receive equal genetic contribution from each parental breed and are expected to retain 50% of heterosis effects [18], though the random process of recombination and segregation generates variance that increases over time around these expectations. However, inbreeding depressions often challenge genetic improvement in practical implementation of synthetic breeding schemes [19, 20].

The simultaneous improvement of animal productivity, adaptability and fitness is imperative for the sustainability of genetic improvement in extensive smallholder production systems. Multi-trait selection strategies enable the simultaneous improvement of both adaptive and productive traits. Selection index methodology allows consideration of specific weights for specific traits, depending on the expected breeding goals, the production environment and the marketing systems [21]. Moreover, optimal trait combinations to prevent unfavourable correlated selection responses strongly depend on the genetic correlations among traits. This implies prior information on trait genetic architecture, which is generally scant for African livestock production systems [22].

The aim of this study is to study effective structured crossbreeding strategies for African smallholder beef cattle systems via comprehensive simulations. Phenotypic and genetic gains, as well as genetic effects (additive and dominance) for both productive and adaptive traits in synthetic crosses were compared with those of F1, two-breed rotational crosses, and pure local animals. Our approach evaluated various genetic correlations between productive and adaptive traits, GxE interactions between local and exotic germplasm and environments, and different selection indexes. Additionally, we assessed the effectiveness of various bull selection methods in synthetic breeding with regard to inbreeding and enhancements of animal performances.

## Materials and methods

**Fig. 1:**
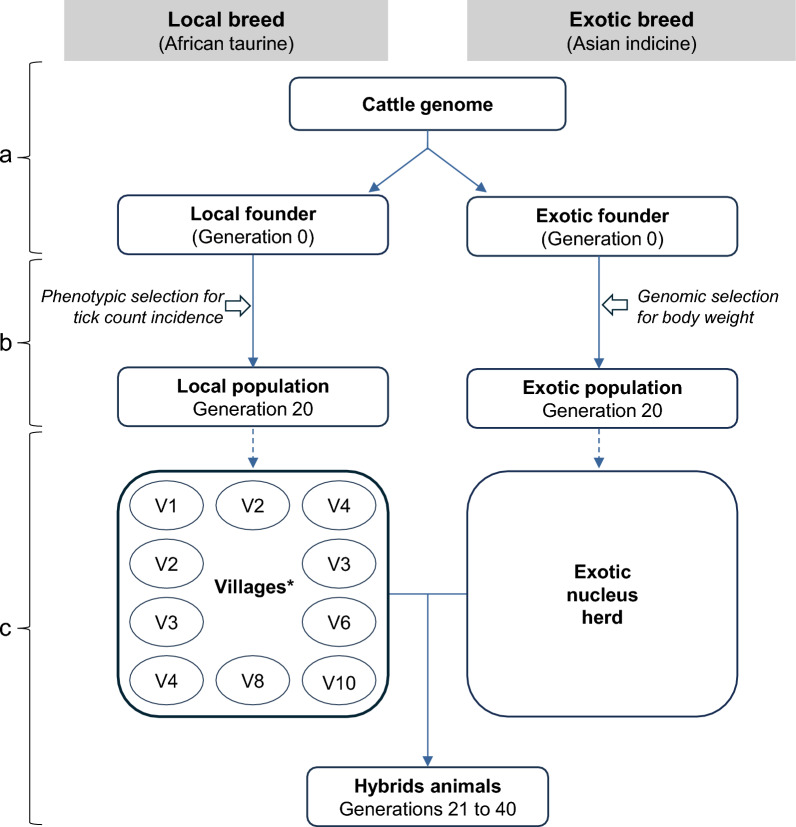
Overview of the simulation pipeline. **a** Simulation of founder cattle genome and complex traits; **b** pure breeding in the local and exotic breed; **c** simulation of smallholder and nucleus herds and design of crossbreeding strategies

Stochastic simulations of crossbreeding programmes were implemented in AlphaSimR [23], following the different steps as outlined in Fig. 1. In brief, we first simulated cattle genomes and polygenic traits for a local breed and an exotic breed. Second, we kept the two breeds separately in pure breeding programs for 20 generations. Third, we created smallholder farms and nucleus herds, and designed different crossbreeding strategies for an additional 20 generations. The crossbreeding strategies were compared via different scenarios. The following sections provide simulation details in this regard, while the simulation scripts are freely available at GitHub (https://github.com/SeyiV/Simulation_Crossbreeding).

### Simulation of founder cattle genome and complex traits

The cattle genome was simulated to mimic a local breed (African taurine) and an exotic breed (Asian indicine). We considered the cattle demography history according to MacLeod et al. [24], and used the Markovian Coalescent Simulator (for details see Chen et al. [25]) with the runMacs function in the AlphaSimR package [23]. As for *Bos taurus* and *Bos indicus*, we assumed that the two breeds shared the same original ancestor, but experienced a historical population split 20,000 generations (100,000 years) ago and we retained 51,000 segregating sites. This strategy led to two genetically distinct founder populations representing the local and the exotic breeds. The average Wright’s Fst value between the two founder populations was 0.41 across all segregating sites. Each founder population was simulated considering 2000 females and 500 males, leading to a total of 5000 founder individuals across two sub-species. The cattle genome comprised 30 chromosome pairs. Each chromosome had 300 quantitative trait loci (QTL) and 1400 single nucleotide polymorphisms (SNP) markers randomly distributed.

We simulated four polygenic traits to represent mature body weight and tick count incidence for a local environment, i.e., Africa (BW_L_ and TC_L_, respectively), and for an exotic environment, i.e., a foreign country (BW_E_ and TC_E_, respectively). Tick count incidence corresponded to $-$log_10_(tick count). We assumed that an animal kept in the local environment has higher adaptive performance (TC_L_), whereas an animal kept in the exotic environment under a structured breeding program has a better productive performance (BW_E_). Existing literature [26–29] was considered to define different phenotypic means and variances and to mimic these differences between the two production environments (Table 1). The four traits shared the same QTL with correlated additive and dominance effects. The genetic correlation (${r}_{g}$) between traits in the same environment, i.e., ${{r}_{g}}_{({BW}_{L},{ TC}_{L})}$ and ${{r}_{g}}_{({BW}_{E},{ TC}_{E})}$, was set to − 0.4, 0, or 0.4 in different scenarios. Furthermore, GxE effects were simulated by assuming a genetic correlation (${r}_{g\times e}$) of 0.4, 0.6 or 0.8 between the same trait expressed in the local and exotic environment, i.e., ${{r}_{g\times e}}_{({BW}_{L},\,{ BW}_{E})}$ and ${{r}_{g\times e}}_{({TC}_{L},\,{ TC}_{E})}$. The values of 0.4, 0.6 and 0.8 corresponded to strong, moderate, and limited GxE effects. QTL effects $({a}_{i})$ were sampled for the four traits from a multivariate normal distribution to achieve the defined ${r}_{g}$, ${r}_{g\times e}$, phenotypic means and genetic variances (Table 1). Dominance effects $({d}_{i})$ at a QTL $i$ were obtained as: ${d}_{i}= {|a}_{i}| \times {\delta }_{i}$; where ${\delta }_{i}$, the dominance degree at the QTL $i,$ was sampled for each trait from a multivariate normal distribution with the means and variances described in Table 1. As a result, we obtained dominance variances in the founder populations and heterosis values in F1 offspring similar to parameters in the literature (see Additional file 1: Tables S1 and S2) [30–32]. Animal phenotypes were simulated by adding a residual environmental effect (random error) to the genetic value for each trait. The random errors were randomly sampled from a normal distribution with a mean of 0 and variance such that the narrow-sense heritability (h^2^) was 0.3 ± 0.03 for BW_L_ and BW_E_, and 0.1 ± 0.01 for TC_L_ and TC_E_ in the respective founder populations. When h^2^ of a trait was outside these ranges in a population, the simulation was discarded.

**Table 1:** Genetic parameters used to simulate the traits in the founder populations

Parameters	Local breed		Exotic breed	
	BW_L_	TC_L_	BW_E_	TC_E_
Narrow-sense heritability (h^2^)	0.3	0.1	0.3	0.1
Phenotypic mean (µ)	325	− 1	450	− 1.5
Phenotypic variance (V_p_)	1300	0.2	625	0.2
Genetic variance (V_a_)	390	0.02	187.50	0.02
Mean of dominance degree	0.2	0.2	0.2	0.2
Variance of dominance degree	1	1.2	1	1.2

BW_L_ = body weight (kg) in the local environment, TC_L_ = tick count incidence in the local environment, BW_E_ = body weight (kg) in the exotic environment, TC_E_ = tick count incidence in the exotic environment. Tick count incidence = − log_10_(tick count); the phenotypic means of − 1 for TC_L_ and − 1.5 for TC_E_ correspond to raw tick counts of 10 and 31, respectively

### Simulation of pure breeding in the local and exotic breed

We generated 20 generations of pure breeding in each breed separately to initiate selection and to create trait and breed specific linkage-disequilibrium beyond the coalescent simulation. In general, each simulated generation comprised 50% males and 50% females, and each cow produced one offspring per mating. We increased the population of the local breed to 10,000 animals per generation, to ensure a sufficient number of local cows for the crossbreeding schemes. In this regard, all simulated local cows were randomly mated with 200 selected local bulls in the first five generations. Afterwards, 10,000 cows were selected and mated with 200 bulls to produce respective local offspring in each generation. Cows were selected within the last five generations and bulls within the last two generations. Animals from the local breeds were selected based on their phenotypic values for TC_L_. This resulted in a phenotypic mean for TC_L_ of 1.00 ± 0.05 in generation 20 (G_20_), with a phenotypic gain of 0.03 per generation. In the exotic breed, the population size comprised 2000 animals per generation. Hence, 2000 cows and 50 bulls were selected and randomly mated to produce progeny for the next generation. Exotic cows and bulls were selected based on their estimated breeding values (EBV) for BW_E_. To mimic past long-term breeding efforts and to generate genetic distance and genetic mean difference between breeds, we used estimated breeding values from the *RR-BLUP* function of AlphaSimR using SNP genotype information and considering all animals from the last five generations. Based on this breeding strategy, the phenotypic mean of BW_E_ in the exotic breed was on average 598.70 ± 7.83 kg in G_20_ with an increase of 7.38 kg per generation. The Additional file 2: Figure S1 describes the changes in phenotypic means for the other traits in response to the selection of TC_L_ and BW_E_ in the local and exotic breed, respectively, depending on ${r}_{g}$ and ${r}_{g\times e}$. Furthermore, the Additional file 3: Table S3 includes genetic parameters (additive genetic variances, heritability, etc.) for all four traits in each breed. The average Wright’s Fst value between the two breeds prior to the crossbreeding schemes (i.e., at G_20_) was 0.3, corresponding to the reported genetic distance between African taurine and Asian indicine by Kim et al. [33].

### Simulation of crossbreeding strategies

In a first step, we simulated a total of 200 smallholder farms, one local nucleus herd and one exotic nucleus herd. All crossbreeding strategies were implemented within the smallholder farms. Eight to 40 local cows were randomly selected from G_20_ to form each smallholder farm, yielding a total population of 4000 to 5000 cows. The smallholder farm sizes were randomly sampled at the start of each replicate, and therefore varied between replicates. Moreover, the smallholder farms were randomly allocated to 10 villages, i.e., 20 farms per village, resulting in variable numbers of animals per village. The simulated farm sizes and villages in a replicate were kept constant throughout the 20 generations and used for all crossbreeding strategies. The local nucleus herd represented a government farm, committed to preserving the local breed for its adaptive traits. We used the nucleus herd to generate purebred local bulls, which were provided to the smallholder farms in the framework of the rotational crossbreeding program (see below). The local nucleus herd was created by selecting 2000 local cows from G_16_ to G_19_. Subsequently, 2000 cows and 50 bulls were mated to produce progenies at each generation. Animals were selected in the local nucleus based on their phenotypic value for TC_L_. The exotic nucleus herd was assumed to be a foreign herd that delivered semen from exotic bulls to the smallholder farms for crossbreeding. The simulated exotic breed (2000 animals per generation) was used to represent this exotic nucleus herd. We continued the same breeding strategy for the exotic breed until G_40_, i.e., by applying genomic selection for BW_E_ to produce exotic progeny for the crossbreeding program.

**Fig. 2:**
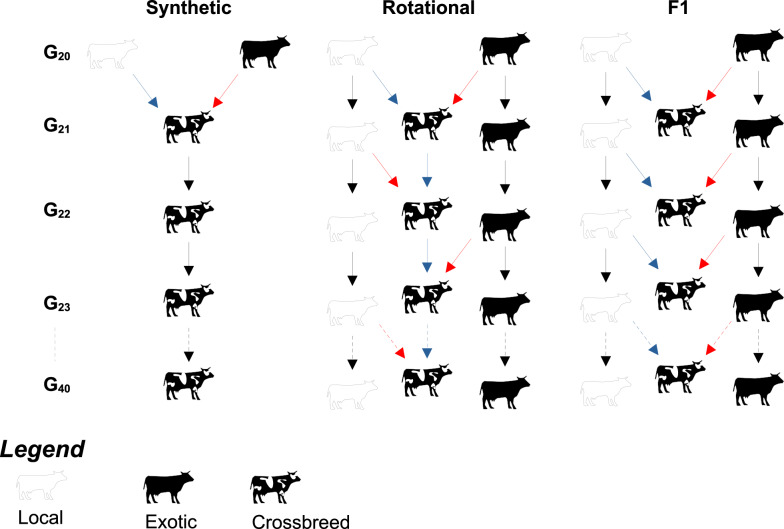
Description of the simulated crossbreeding strategies. Black arrow = random mating of selected sires and dams within population, blue arrow = use of selected dams, red arrow = use of selected sires. For synthetic breeding, all animals were produced within the smallholder farms. With the rotational strategy, the crossbred cows were selected in smallholder farms, while the local and exotic sires were selected from the local and exotic nucleus herds respectively. The F1 crossing involved the production and selection of local animals on the smallholder farms, and the selection of exotic sires from the exotic nucleus herd

Separate simulation procedures were applied to mimic F1 and rotational crossbreeding as well as synthetic breed formation (Fig. 2). For all crossbreeding strategies, we produced a first generation of crossbred animals (G_21_) by mating local cows from the smallholder farms with exotic bulls. In this regard, fifty exotic bulls were selected at G_20_ and assigned to the 10 villages, implying that the semen of a bull was used in four smallholder farms.

With regard to synthetic breeding, crossbred cows and bulls were selected and mated to produce offspring at each generation. We simulated over 19 subsequent generations for four synthetic breeding schemes, which were defined as farm bull (FB), intra-village bull (IVB), exchanged-village bull (EVB), and population-wide bull (PWB). The four synthetic breeding schemes differed in the selection of crossbred bulls and their farm allocations. Regarding FB, one crossbred bull was selected per farm and mated with cows from the same farm. The FB scheme implied closed matings of relatives at the farm level, and mimicked a practice commonly observed in many smallholder farms in Africa. The IVB scheme assumed a cooperation between the smallholder farms with exchanges of bulls within the same village. Hence, the five best bulls in a village ${v}_{i}$ were selected and randomly allocated to the farms located in the same village (${v}_{i}$). The EVB scheme enabled the exchange of bulls among villages. In this regard, five bulls were selected in a village ${v}_{i}$ and randomly assigned to the farms located in another village ${v}_{j}$. The village ${v}_{j}$ was randomly chosen at each generation but differed from ${v}_{i}$. In the PWB scheme, all candidate bulls from the 200 smallholder farms were pooled together. The 50 best bulls were selected and randomly assigned to the farms regardless of their initial farm and village origin. Consequently, in contrast to the other synthetic schemes, the number of bulls selected per village in PWB was variable.

Rotational crossbreeding was also simulated for another 19 generations (G_22_ to G_40_). In this regard, crossbred cows from the smallholder farms were mated with local bulls at even generations, and with exotic bulls at odd generations. Fifty bulls were selected from the last two generations either of the local nucleus herd or of the exotic nucleus herd, and five of these bulls were randomly assigned to each village.

F1 crosses were also simulated over 19 additional generations. We kept a pure line of local animals in each smallholder farm, in parallel to the crossbreeding scheme. In this regard, we exceptionally assumed that each local cow produced two progenies per generation in this strategy. First, local cows (C_n_) were mated with exotic bulls to produce F1 offspring at each generation (G_n_). The exotic bulls were selected from G_n−1_ and G_n−2_ in the exotic nucleus herd and randomly assigned to the 10 villages (one bull for four smallholder farmers). None of the F1 offspring were used as breeding animals, because all were slaughtered for meat production. Second, the same local cows (C_n_) were mated with local bulls to produce a local progeny for G_n_. The reason of this approach is to ensure the availability of local cows at each generation for ongoing production of F1 animals on the smallholder farms. The local cows and bulls were selected from the local progenies based on their phenotypic value for TC_L_ to serve as parents for the next generation. Five local bulls were selected from G_n−1_ and G_n−2_ in each village and randomly assigned to the farms, while the local cows were selected within G_n−1_ to G_n−5_ populations at the farm level. The generated local offspring on the smallholder farms formed the local breed from G_21_ to G_40_ and were compared with the different crosses.

We used a multi-trait selection index to select crossbred bulls and cows for the synthetic and the rotational breeding strategies. We only considered phenotypic values due to the limitations of animal genotyping in African smallholder farms [11, 34]. Hence, the selection index was defined for each animal as a linear combination of its phenotypic values for BW_L_ and TC_L_, weighted by trait specific economic values. The economic value of BW_L_ was set to one. For TC_L_, we simulated different economic values to achieve the relative breeding goal emphasis of 10%, 30% and 50%, leading to three selection index ($SI$) scenarios (SI_TCL10%, SI_TCL30%, SI_TCL50%, see Table 2). The economic values for SI_TCL10% corresponded to those reported by Simões et al. [35]. The relative emphasis of TC_L_ (${e}_{{TC}_{L}}$) was calculated following Santos et al. [36] as ${e}_{{TC}_{L}}={(\sigma }_{{TC}_{L}}.{v}_{{TC}_{L}})\times 100/{(\sigma }_{{TC}_{L}}.{v}_{{TC}_{L}}+{\sigma }_{{BW}_{L}})$; where ${\sigma }_{{BW}_{L}}$ and ${\sigma }_{{TC}_{L}}$ were the phenotypic standard deviation for BW_L_ and TC_L,_ respectively, and ${v}_{{TC}_{L}}$ was the economic value for TC_L_.

**Table 2:** Economic weights for breeding goal traits used in selection indexes

Selection index	Economic value (in Euro)		Relative emphasis	
	BW_L_	TC_L_	BW_L_ (%)	TC_L_ (%)
SI_TC_L_10%	1	10	90	10
SI_TC_L_30%	1	35	70	30
SI_TC_L_50%	1	80	50	50

### Scenarios, replicates, evaluation parameters and statistical analyses

The combinations of the different ${r}_{g}$, ${r}_{g\times e}$, and $SI$ implied a total of 27 different scenarios (Table 3). Each scenario was replicated 40 times. In one replicate, we run all crossbreeding strategies using the same starting populations from G_20_. We focused on the traits BW_L_ and TC_L_ to evaluate the crossbreeding strategies and scenarios. The phenotypic mean, additive genetic variance and average genomic inbreeding coefficient were calculated per generation simulated during a replicate. The genomic inbreeding coefficient ($F$) of an individual was calculated following Werner et al. [37] as $F={SNP}_{hom}\times 100$ / ${SNP}_{tot}$, where ${SNP}_{hom}$ and ${SNP}_{tot}$ were the number of homozygous SNP and total number of SNP, respectively. Moreover, true breeding values (TBV) and dominance deviations (DD) were calculated by merging all animals from G_21_ to G_40_ produced by a crossbreeding strategy in a replicate. The TBV or DD of an animal corresponded to its respective deviation from the mean value of the population of the 20 generations. We averaged the TBV and DD per generation to evaluate respective trends for each crossbreeding strategy. Furthermore, phenotypic and genetic gain were calculated as the regression slope of the phenotypic mean and average TBV, respectively, on the generations. For the final reports, all calculated parameters were averaged over the 40 replicates.

**Table 3:** Overview of the parameters used to generate different breeding scenarios

Scenario parameter	Value	Comment
Genetic correlation $({r}_{g})$	− 0.4	Negative genetic correlation between body weight and tick count incidence in each environment
	0	No genetic correlation between body weight and tick count incidence in each environment
	0.4	Positive genetic correlation between body weight and tick count incidence in each environment
GxE effects $({r}_{g\times e})$	0.4	Strong GxE effects for both traits between local and exotic environment
	0.6	Moderate GxE effects for both traits between local and exotic environment
	0.8	Limited GxE effects for both traits between local and exotic environment
Selection index $(SI)$	SI_TC_L_10%	Economic weighting schemes (see Table 2)
	SI_TC_L_30%	
	SI_TC_L_50%	

## Results

### Crossbreeding performances for uncorrelated traits under moderate GxE effects

**Fig. 3:**
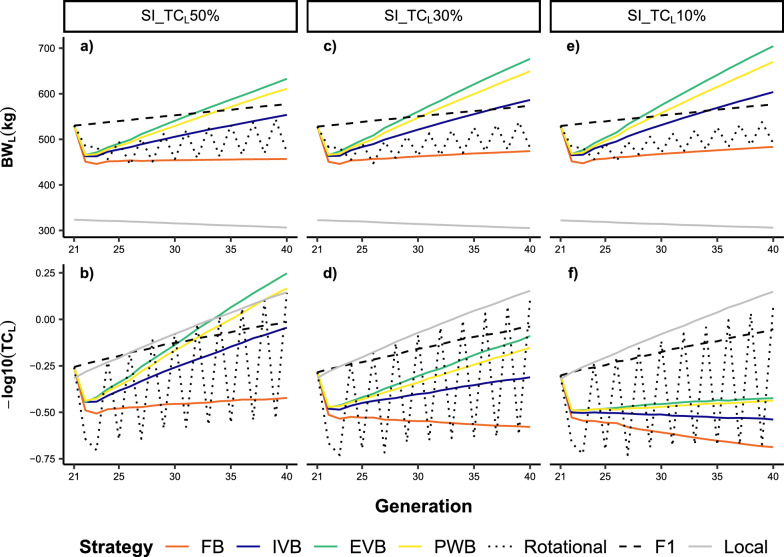
Phenotypic values for body weight (BW_L_) and tick count incidence (TC_L_) over 20 generations in different crossbreeds and local cattle populations simulated on smallholder farms. Different selection index scenarios were evaluated (see Table 2 for more details): **a** BW_L_ with SI_TC_L_50%; **b** TC_L_ with SI_TC_L_50%; **c** BW_L_ with SI_TC_L_30%; **d** TC_L_ with SI_TC_L_30%; **e** BW_L_ with SI_TC_L_10%; **f** TC_L_ with SI_TC_L_10%. A genetic correlation between BW_L_ and TC_L_ of 0, and genetic-by-environment interaction between local and exotic environments of 0.6 were assumed. FB = farm bull synthetic scheme, IVB = intra-village bull synthetic scheme, EVB = extra-village bull synthetic scheme, PWB = population-wide bull synthetic scheme

**Fig. 4:**
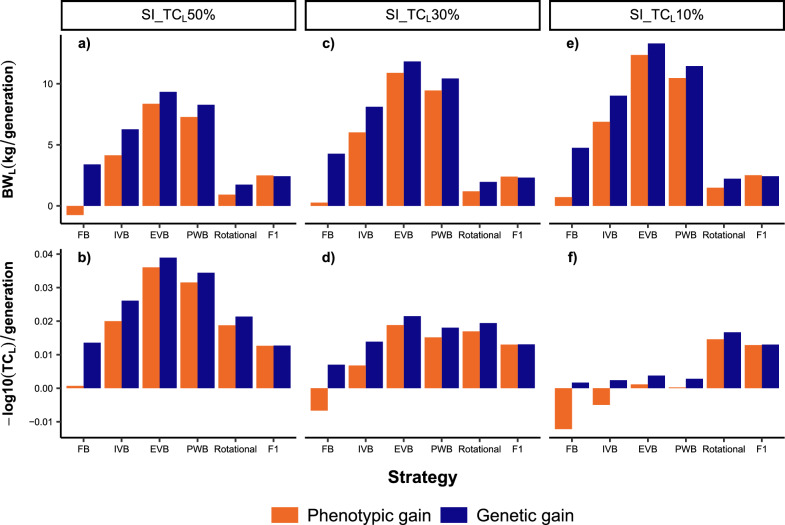
Phenotypic and genetic gains expressed as the regression coefficients of phenotypic and true breeding values, respectively, for body weight (BW_L_) and tick count incidence (TC_L_) over 20 generations in different crossbreed cattle populations simulated on smallholder farms. Different selection index scenarios were evaluated (see Table 2 for more details): **a** BW_L_ with SI_TC_L_50%; **b** TC_L_ with SI_TC_L_50%; **c** BW_L_ with SI_TC_L_30%; **d** TC_L_ with SI_TC_L_30%; **e** BW_L_ with SI_TC_L_10%; **f** TC_L_ with SI_TC_L_10%. A genetic correlation between BW_L_ and TC_L_ of 0, and genetic-by-environment interaction between local and exotic environments of 0.6 were assumed. FB = farm bull synthetic scheme, IVB = intra-village bull synthetic scheme, EVB = extra-village bull synthetic scheme, PWB = population-wide bull synthetic scheme

This section describes the results of the simulated crossbreeding strategies for the scenarios with ${r}_{g}$ = 0 and ${r}_{g\times e}$= 0.6. We first focus on the results for SI_TC_L_50%, and present afterwards the differences compared to the scenarios SI_TC_L_30% and SI_TC_L_10%. Figures 3a and b displays the evolution of the phenotypic means for BW_L_ and TC_L_ in the crossbred and local populations over 20 generations. All crossbreed populations showed significantly higher phenotypic means for BW_L_ than the local breed. Inversely, the phenotypic means of TC_L_ were higher in F1 crosses compared to the local breed between G_21_ and G_26_, but not afterwards. More interestingly, the EVB crosses outperformed the local breed in terms of the phenotypic mean for TC_L_ from G_34_. The synthetic crosses indicated a significant reduction in phenotypic means for both traits at G_22_. However, there was a substantial increase in the phenotypic means of BW_L_ and TC_L_ in subsequent generations, particularly in EVB and PWB. These two schemes delivered higher phenotypic means for BW_L_ than rotational and F1 crosses from G_26_ and G_34_, respectively. Similarly, EVB and PWB crosses outperformed F1 crosses regarding the phenotypic means of TC_L_ from G_31_ and G_32,_ respectively. Rotational crossing implied fluctuating phenotypic means across generations. The use of exotic bulls led to high phenotypic means for BW_L_ in rotational crosses, but with low phenotypic means for TC_L_. Inverse results were observed in rotational generations from local bulls. Over the 20 generations, EVB, PWB and IVB generated higher phenotypic gains for both traits than F1 and rotational crosses (Fig. 4). The highest gains were obtained in EVB, and the lowest in FB. The application of FB implied a negative phenotypic gain, which contrasted with its positive genetic gain over the 20 generations. Indeed, phenotypic gains were smaller than genetic gains in all synthetic breeding schemes and for rotational crosses, being in contrast with the results from the F1 crosses (Fig. 4). Moreover, all synthetic crosses displayed larger genetic gains than F1 and rotational crosses.

**Fig. 5:**
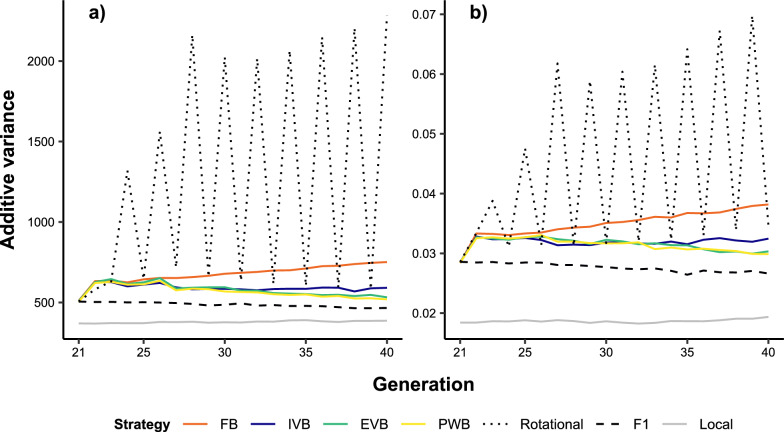
Additive genetic variance for **a** body weight (BW_L_) and **b** tick count incidence (TC_L_) over 20 generations in different crossbreed and local cattle populations simulated on smallholder farms. A genetic correlation between BW_L_ and TC_L_ of 0, and genetic-by-environment interaction between local and exotic environments of 0.6 were assumed. In addition, the selection index SI_TC_L_50% was applied (see Table 2 for more details). FB = farm bull synthetic scheme, IVB = intra-village bull synthetic scheme, EVB = extra-village bull synthetic scheme, PWB = population-wide bull synthetic scheme

**Fig. 6:**
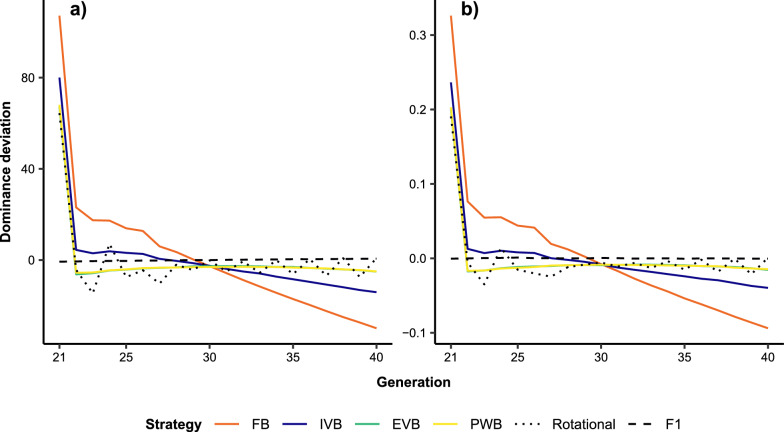
Dominance deviation for **a** body weight (BW_L_) and **b** tick count incidence (TC_L_) over 20 generations in different crossbreed cattle populations simulated on smallholder farms. A genetic correlation between BW_L_ and TC_L_ of 0, and genetic-by-environment interaction between local and exotic environments of 0.6 were assumed. In addition, the selection index SI_TC_L_50% was applied (see Table 2 for more details). FB = farm bull synthetic scheme, IVB = intra-village bull synthetic scheme, EVB = extra-village bull synthetic scheme, PWB = population-wide bull synthetic scheme

**Fig. 7:**
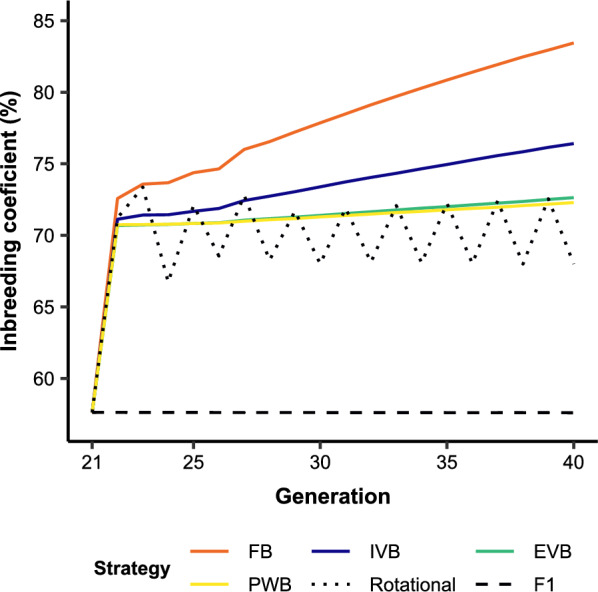
Inbreeding coefficient over 20 generations in different crossbreed cattle populations simulated on smallholder farms. A genetic correlation between BW_L_ and TC_L_ of 0, and genetic-by-environment interaction between local and exotic environments of 0.6 were assumed. In addition, the selection index SI_TC_L_50% was applied (see Table 2 for more details). FB = farm bull synthetic scheme, IVB = intra-village bull synthetic scheme, EVB = extra-village bull synthetic scheme, PWB = population-wide bull synthetic scheme

Additive genetic variances for BW_L_ and TC_L_ were significantly higher in the crossbreed populations than in the local breed as expected (Fig. 5). The rotational and synthetic crosses delivered the highest additive genetic variances for both traits from G_22_ to G_40_. In contrast to FB, there was a slight decrease in additive genetic variances in EVB, PWB and IVB after G_26_. Significant reductions in dominance deviations for both traits were observed in all synthetic crosses at G_22_ (Fig. 6). From G_23_, dominance deviations in EVB and PWB decreased only slightly and were similar. Conversely, FB crosses showed the largest reduction of dominance deviations across the 20 generations. Decreases in dominance deviation were associated with increases in inbreeding coefficients as mainly observed at G_22_ with the synthetic breeding schemes (Fig. 7). Inbreeding increased significantly and continuously from G_21_ to G_40_ in IVB and FB. The FB scheme induced the largest increase of inbreeding coefficient from 57.5% at G_1_ to 83.3% at G_20_. Inbreeding coefficients in PWB and EVB did not differ from each other and were lower than for FB and IVB. In general, inbreeding coefficients of the synthetic breeding schemes were slightly higher than in rotational crosses but were much larger when compared with F1 crosses.

The use of SI_TC_L_30% and SI_TC_L_10% implied lower phenotypic gain for TC_L_, with a corresponding increase in phenotypic gains for BW_L_ in all synthetic breeding schemes (Fig. 4). The EVB crosses still delivered the highest phenotypic and genetic gains for both traits when SI_TC_L_30% is applied. However, the phenotypic means for TC_L_ at G_40_ were lower in the EVB crosses compared to F1 crosses (Fig. 3d). With regard to SI_TC_L_10%, phenotypic gains for TC_L_ were nearly zero in EVB and PWB, and negative in FB and IVB crosses (Fig. 4f).

### Crossbreeding performances for correlated traits and impacts of GxE effects

**Fig. 8:**
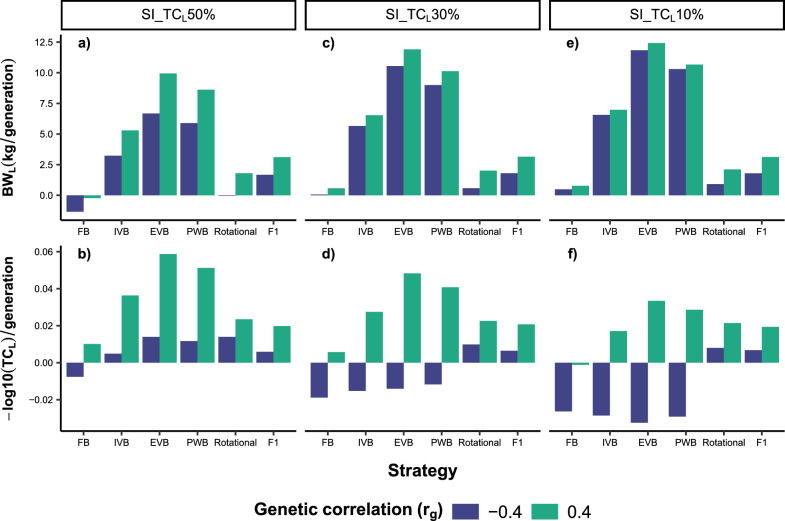
Difference in phenotypic gain for body weight (BW_L_) and tick count incidence (TC_L_) over 20 generations in different crossbreed cattle populations due to genetic correlations between the two traits and applied selection index (see Table 2 for more details): **a** BW_L_ with SI_TC_L_50%; **b** TC_L_ with SI_TC_L_50%; **c** BW_L_ with SI_TC_L_30%; **d **TC_L_ with SI_TC_L_30%; **e** BW_L_ with SI_TC_L_10%; **f** TC_L_ with SI_TC_L_10%. A genetic-by-environment interaction between local and exotic environment of 0.6 was assumed FB = farm bull synthetic scheme, IVB = intra-village bull synthetic scheme, EVB = extra-village bull synthetic scheme, PWB = population-wide bull synthetic scheme

First, we compare the results of the scenarios with ${r}_{g}$ = 0.4 and ${r}_{g}$ = − 0.4, while ${r}_{g\times e}$= 0.6 and SI_TC_L_50% was fixed. Phenotypic means, phenotypic gains and genotypic gains for both traits were generally higher with ${r}_{g}$ = 0.4 than with ${r}_{g}$ = − 0.4. Overall, the crossbreeding strategies led to higher phenotypic means for BW_L_ when compared to that in the local breed, irrespective of ${r}_{g}$ (See Additional file 4: Figure S2). For ${r}_{g}$ = 0.4, the phenotypic means for TC_L_ in F1 and EVB crosses were higher than in the local breed over the entire crossbreeding period. Inversely, significantly lower phenotypic means of TC_L_ were generally obtained in all crossbred populations compared to the local breed with ${r}_{g}$ = − 0.4. Among the crossbreed populations, EVB exhibited the highest phenotypic gains for both traits irrespective of the ${r}_{g}$ (Fig. 8a and b). EVB implied higher phenotypic means for BW_L_ than F1 crosses from G_30_ with ${r}_{g}$ = 0.4, and from G_35_ with ${r}_{g}$ = − 0.4 (see Additional file 4: Figure S2). Another main difference related to ${r}_{g}$ addresses the evolution of the phenotypic means for TC_L_. With ${r}_{g}$ = 0.4, EVB, PWB and IVB implied higher phenotypic means for TC_L_ than F1 crosses already from G_27_, G_28_ and G_31_, respectively. With ${r}_{g}$ = − 0.4, F1 crosses had higher phenotypic means of TC_L_ over the 20 generations compared to the synthetic animals, but the difference with the phenotypic means of TC_L_ in EVB and PWB crosses at G_40_ was not significant. Moreover, inter-generational differences in phenotypic means for both traits were larger when applying rotational crossing and assuming ${r}_{g}$ = − 0.4 (see Additional file 4: Figure S2).

Changing the breeding goal to SI_TC_L_30% and SI_TC_L_10% led to decreasing phenotypic means for TC_L_ over the 20 generations with ${r}_{g}$ = − 0.4 (Fig. 8d and f, see Additional file 4: Figure S2d and S2f). With ${r}_{g}$ = 0.4, increased phenotypic means for BW_L_ and TC_L_ in the 20 generations were only observed for the EVB, PWB and IVB crosses, regardless of the $SI$ variant.

GxE effects decreased the phenotypic means for BW_L_ in all crossbreed populations, irrespective of ${r}_{g}$. The presence of strong GxE effects (${r}_{g\times e}$= 0.4) resulted in the lowest phenotypic means for BW_L_ compared to ${r}_{g\times e}$= 0.8 and ${r}_{g\times e}$ = 0.6 (see Additional file 4: Figure S3). Similar results were observed for TC_L_ with ${r}_{g}$ = 0.4. However, the presence of strong GxE effects (${r}_{g\times e}$= 0.4) led to slightly higher phenotypic means for TC_L_ in the crossbred populations with ${r}_{g}$ = − 0.4. The synthetic breeding schemes displayed no significant differences in phenotypic and genetic gains due to ${r}_{g\times e}$, unlike F1 and rotational crosses. The EVB and PWB crosses had the highest phenotypic means for BW_L_ at G_40_, irrespective of the ${r}_{g\times e}$ values.

## Discussion

The overall results indicate greatest potential for EVB and PWB schemes with synthetics to promote durable genetic improvement for both productive and adaptive traits in smallholder cattle farming contexts. More specifically, EVB and PWB exhibited the highest phenotypic mean for BW_L_ at G_40_, and the highest phenotypic and genetic gains for BW_L_ over the 20 generations, regardless of ${r}_{g}$ and ${r}_{g\times e}$. A relative emphasis of 50% on TC_L_ produces similar results for TC_L_. However, a lower emphasis (SI_TCL10% and SI_TC_L_30%) limits phenotypic and genetic gains for TC_L_ in the EVB and PWB crosses in case of negative ${r}_{g}$ or ${r}_{g}$ equal to 0. These findings outline three key points for the discussion: (1) long-term genetic improvement and phenotypic stability of crossbred populations in smallholder farms; (2) simultaneous improvement of adaptive and productive traits in presence of unfavourable genetic correlations and GxE effects; and (3) practical implications for genetic improvement programs in African smallholder farms. We also mention limitations of this study.

### Long-term genetic improvement and phenotypic stability of crossbred populations in smallholder farms

The higher genetic gain for BW_L_ and TC_L_ over the 20 generations in EVB and PWB schemes, with SI_TC_L_50% irrespective of ${r}_{g}$ and ${r}_{g\times e}$, underlines the ability of these schemes (a) to capitalize on the higher genetic variances of synthetic crosses to generate high responses to long-term selection, and (b) to retain heterosis in advanced generations of synthetic animals. The crossing of local and exotic breeds increases additive genetic variance for both traits in G_21_ because of the differences in their QTL allele frequencies [38]. These larger additive genetic variances enable greater response to selection in subsequent generations of synthetic crosses. Inversely, genetic gain in F1 crosses over the 20 generations depends on the respective ongoing performances of the exotic and local breeds. Decreasing genetic variances due to selection, and fewer genetic improvement in the exotic and local breeds, explain the observed lower genetic gains in the F1 crosses over the 20 generations. Our findings corroborate with previous reports indicating the advantages and greater potential for improvement of synthetic animals compared to original breeds [39, 40]. Also, the results are consistent with the success of several synthetic or composite crossbreeds like Brangus in a worldwide context [41, 42].

The fluctuation of phenotypic means between generations in rotational crosses is caused by the phenotypic and genetic differences for both traits between exotic and local breeds. Ongoing genetic improvement and negative correlation between the traits enhance these differences, inducing larger fluctuations in later generations. Our results are consistent with previous findings evaluating the performance of rotational crossbreeding [43, 44]. The intergenerational phenotypic and genetic divergences in rotational crosses imply high variability of animal performances in smallholder herds in terms of productive and adaptive characteristics. This absence of phenotypic stability has a low chance to sustain in smallholder production systems, because of the required diverse management strategies depending on the requirements of heterogeneous animal groups. Gregory and Cundiff [41] noted the unsuitability of rotational crossbreeding in the case of large breed differences (as between local and exotic breeds in developing countries). This observation justifies the frequent application of rotational crossbreeding in programs involving only similar populations like temperate breeds [45–47].

The observed differences between genetic and phenotypic gain over the respective 20 generations in F1, rotational, and synthetic crosses are due to the presence of dominance effects. The differences are associated with the evolution of inbreeding coefficients causing inbreeding depression and heterosis. The accumulation of homozygous alleles in highly inbred animals increase the expression of deleterious recessive alleles, leading to inbreeding depression [48, 49]. Inversely, low inbreeding coefficient favour heterozygous loci to express heterosis and possibly even overdominance [49, 50]. Hence, the superior phenotypic gain over genetic gain in the F1 crosses across 20 generations is due to independent selection within the local and exotic population, which then maximised heterosis of F1 crosses [48]. In contrast, lower phenotypic gain compared to genetic gain in the synthetic and rotational crosses suggest presence of inbreeding depression from G_22_. Indeed, the significant increase in inbreeding coefficients at G_22_ in the synthetic and rotational crosses causes significant decreases in dominance deviations, which consequently leads to the observed decrease in the phenotypic means for BW_L_ and TC_L_. These results concur well with previous reports on the loss of heterosis in F2 and rotational crosses [41]. The loss of heterosis in G_22_ is responsible for the lower phenotypic means of the synthetic crosses in the first five generations (irrespective of the scenarios), and confirms the need of several generations to achieve improved performances in synthetic crosses [39].

The FB crosses experience a significant increase in inbreeding coefficients from G_23_ to G_40_, and in consequence, are most affected by inbreeding depression. This explains the negative phenotypic trend over the 20 generations in some scenarios despite their positive genetic gains. Exchanges of bulls in IVB, EVB, and PWB enable a significant control of inbreeding. The quite stable inbreeding coefficient from G_22_ to G_40_ in PWB crosses demonstrates the importance of a larger population to maintain genetic diversity in synthetic animals. Similar inbreeding coefficient in EVB crosses concurs well with the effectiveness of circular breeding schemes to lower inbreeding in early and advanced generations [51]. The observed correlation between low inbreeding and quite stable dominance deviations from G_23_ to G_40_ suggests that EVB and PWB limit the expression of unfavourable recessive alleles by retaining heterozygous alleles in advanced generations. Evidence of retained heterosis in advanced generations of synthetic crosses of cattle has been similarly reported in previous studies [40, 52].

High genetic and phenotypic gains in EVB and PWB in contrast to FB confirm previous observations that lowering inbreeding is a key driver of genetic improvement in synthetic breeding [53]. Population-wide inbreeding reduces genetic diversity and therefore response to selection. The poor phenotypic and genetic gains of FB crosses for the two traits over the 20 generations indicate the need to avoid selecting farm bulls in smallholder production systems, advocating structured breeding programs [54]. More interestingly, the greater phenotypic and genetic gains in EVB compared to PWB is in line with previous findings reporting higher genetic progress by applying within-family selection compared to individual or mass selection [55, 56]. Walsh and Lynch [57] noted that within-family selection outperforms mass selection for low heritable traits or in presence of large family or environmental effects. Under such circumstances, within-family selection enhances the selection accuracy of traits compared to mass selection, because phenotypic differences between individuals sharing the same environment are more correlated with differences in their breeding value [57]. Therefore, EVB as suggested in this study may be the most appropriate breeding strategy in the context of African smallholder farming. Indeed, the use of genomic selection is limited in Africa while the dominant smallholder farming systems associated with high differences in socio-ecological factors between villages or regions contribute to increased breed diversity [12, 33, 34, 58]. Moreover, EVB can be especially significant in terms of long-term genetic improvement in a crossbreeding program with limited population size. As observed by Dempfle [59], the equal contribution of bulls from each village as implemented via EVB increases the effective population size and selection limits when compared to mass selection (as in PWB). Montaldo [60] demonstrated that the selection response of a trait increases with the number of testing environments and the variability between these environments. This observation suggests a higher number of villages or regions involved in an EVB scheme is expected to increase the genetic gain.

### Simultaneous improvement of adaptive and productive traits in presence of unfavourable genetic correlations and GxE effects

The benefit of crossbreeding in improving productive traits is confirmed by the increase in phenotypic means for BW_L_ in all crosses, regardless of ${r}_{g}$ and ${r}_{g\times e}$. However, changes in the phenotypic means for TC_L_ in crossbred populations due to ${r}_{g}$ highlight the importance to consider genetic correlations between breeding goal traits to prevent deteriorated adaptive performances in crossbred cattle. Results with ${r}_{g}$ = 0.4 indicate the possibility to improve positively correlated traits in a selection index without an antagonistic selection response. On the other hand, the observed phenotypic gains for TC_L_ in EVB and PWB crosses with SI_TC_L_50% demonstrate the possibility of improving both adaptive and productive traits in synthetic populations, also in the presence of unfavourable genetic correlation. Several adaptive or fitness traits (e.g., disease resistance) are unfavourably correlated with productive traits (e.g., body weight) [26, 61, 62]. The decline in phenotypic means for TC_L_ in index scenarios SI_TC_L_10% and SI_TC_L_30% when ${r}_{g}$= − 0.4 indicates that it is imperative to focus breeding on performance and adaptive traits.

A breeding balance among productivity and adaptability might be a challenge, but in an African context, sustained or even improved adaptability is of paramount importance [16, 63]. Improved animal adaptability is associated with robustness and stability and will support the sustainability of smallholder cattle production systems. Moreover, in the case of ${r}_{g}$= − 0.4, the lower phenotypic means of TC_L_ in all crossbred populations including F1 crosses compared to the local breed suggest that an improvement of management along with crossbreeding efforts might be essential to reduce the effects of declining adaptive performance in crossbreds, especially in the first generations [64]. These results corroborate previous reports on the deterioration of adaptive traits in crossbred animals [65]. These results and past studies indicate why many crossbreeding attempts have failed in the context of smallholder settings, where a rapid and substantial change in management might not be feasible. Often-practiced indiscriminate selling of exotic semen and corresponding crossing is therefore highly problematic and not a “quick-fix” solution.

To infer clear differences and mechanisms of selection, our study assumes that the local breed is only selected and improved for its adaptive performance. However, in practical breeding, also productivity improvements play a role, and a genetic improvement focusing on both productive and adaptive traits will similarly lead to declining adaptive performances in the local breed when ${r}_{g}$ is negative. Another approach to genetically improve both adaptive and productive traits might be the selection of synthetic bulls and cows with different breeding goals or selection indexes. A previous study reported greater progress when the bulls are selected for productive traits and the cows for an index of productive and maternal traits [39]. In the context of smallholder breeding systems where animal adaptation is of great importance, synthetic cows may be selected solely for their adaptive traits, while the bulls would be selected for an index of productive and adaptive traits.

The observed impacts of GxE effects on the crossbreed phenotypic values are due to the reranking of the breeding values of the exotic bulls introduced in the local environment [66, 67]. Falconer [68] associated positive selection (upwards) of a trait in a high environment with a negative selection (downwards) in the low environment. This concept of synergistic selection explains the lower phenotypic means for BW_L_ in exotic bulls, and consequently in the crossbred animals when ${r}_{g\times e}$ = 0.4. With positive ${r}_{g}$, the selection for higher BW_E_ in the exotic bulls also implies the upward (synergistic) selection for TC_E_, and therefore similar decrease in the phenotypic mean for TC_L_ in crossbreds in the local environment. However, with negative ${r}_{g}$, the selection for higher BW_E_ implies negative selection for TC_E_ in the exotic bulls. The downward selection for TC_E_ in the exotic environment is associated with upward selection in the local environment [68]. This indirect antagonistic selection for TC_E_ is responsible for observed higher phenotypic means for TC_L_ in the crossbred animals when ${r}_{g}$= − 0.4 and ${r}_{g\times e}$ = 0.4. These findings suggest that higher adaptive performance can be achieved in the presence of unfavourable genetic correlations and GxE effects.

In contrast to F1 and rotational crosses, GxE does not significantly affect phenotypic gains in synthetic crosses because of the direct breeding of synthetic bulls in the local environment. The results are in line with a previous study indicating significant sire GxE effect on offspring performance [69]. In addition, Menéndez-Buxadera et al. [70] reported higher GxE effects with Holstein purebred compared to Holstein-Zebu crossbred. These findings highlight an additional advantage of using synthetic breeding in the African smallholder cattle production environment.

### Practical implications for genetic improvement programs in African smallholder farms

The effectiveness of genetic improvement programs depends on genetic, economic and operational factors [1, 39]. In terms of genetic factors, we discussed above that the formation of a synthetic breed using the EVB scheme is the most appropriate strategy to ensure long-term genetic gains for both adaptive and productive traits. In addition, synthetic breeding based on a selection index to balance traits that improve both animal productivity and adaptability to local conditions, will contribute to farm economy [1], with positive effects on smallholder welfare. Finally, synthetic breeding is the most practical strategy in the smallholder African farming context because of the continuous production and availability of both sexes, i.e., male and female breeding stock in the smallholder farms. The establishment of nucleus herds to continuously produce breeding animals is a major limitation for crossbreeding in most developing countries [5]. The steady production of local replacement females for F1 crossing in smallholder herds is almost unfeasible due to the small herd size. Furthermore, regular import of exotic semen at each generation implies high acquisition costs, which limits ongoing production of F1 animals in commercial farms [45]. Most important for a sustainable implementation, smallholder settings are often unable to cope with the F1 strategy due to the aforementioned challenges. In contrast, the import of exotic semen is only required at the beginning of the synthetic breeding program.

Furthermore, the direct production of breeding males and females in smallholder farms implies some favourable economic aspects when compared to other breeding strategies. The costs of the breeding program could be significantly reduced, because there is no need to create and maintain a nucleus herd for breeding stock production or to regularly acquire costly exotic semen [10]. The lower costs of synthetic breeding will support its durability and sustainability, given that the lack of financial resources is a major constraint for breeding programs in developing countries [1, 17]. From our perspective and experiences, the primary operating costs in synthetic breeding are related to the overall management of breeding programs, animal selection, and the organization of artificial inseminations. Implemented structured management strategies might help to cover the costs of a breeding program. For instance, improved animal productivity associated with well-organized value chains, implying higher income to farmers, can facilitate financial contributions from farmers through membership fees [1]. Alternatively, each local farmer could contribute to the breeding program through genetic contributions from own farm bulls. The best breeding bulls will be selected, and their semen relocated to the farms participating at a community-based breeding program (CBPB). In such context, scientists from local research institutes will support the breeding program through genetic evaluations and the development of mating and selection schemes [12, 34]. Finally, government extension services can assist in managing or supervising the breeding program and in practical operations, such as artificial inseminations or health management. Logistic aspects might hamper the successful implementation of synthetic breeding schemes in African smallholder farms, but there are possibilities to overcome some of these limitations. Also, in the context of logistic challenges, the concept of CBBP has been recommended by researchers to remediate the lack of functional breeder association in African countries [17, 71]. CBBP favours the active participation of farmers in the design and implementation of breeding programs. This guarantees not only the development of suitable animals for local environments, but also the durability of the programs. Moreover, active participation of farmers could facilitate phenotypic data recording as a base for reliable animal selection as in CBBP [61]. Appropriate methods for optimal data collection in small-scale farms could follow existing and successful experiences in some developing regions [72, 73]. Finally, strategies should be developed in the context of CBBP to enhance the adoption of artificial insemination in smallholder cattle farms. Artificial insemination will facilitate the cost-effective exchange of semen and support the management of inbreeding. The increase of technical capacities and infrastructures for artificial insemination in rural areas is a pre-requisite in this regard [12].

In contrast to CBBP, the EVB synthetic breeding approach proposed in this study argues against the limitation of the program to a small community of smallholder farmers [17]. The low performance of the IVB crosses supports this view. Nevertheless, several breeder communities could be organised and collaborate to promote the exchanges of semen following the EVB scheme. More interestingly, collaboration between different regions and countries would be more effective as it will increase the effective population size and animal genetic diversity for better animal adaptability and productivity, while also promoting exchange of knowledge among farmers. Such an initiative is currently ongoing across parts of Africa with the Africa Asia Dairy Genetic Gains (AADGG) programme [74]. Lastly, initial emphasis should be on phenotyping and phenotype-based selection coupled with correction for environmental and management effects, possibly even pedigree-based BLUP. With the continued decreasing cost in SNP array genotyping, prospects for genomic selection in developing countries is rapidly increasing as indicated by the AADGG programme, provided that sufficient support is provided in the initial stages of setting up such genomic programmes. Once such genomic information will be available, the synthetic strategy could be augmented by differentiating the origin and effect of alleles in synthetic animals [75–77] to facilitate more accurate selection of local animals and more appropriate import of exotic animals.

### Replicability and limitations of the study

The consistent differences in phenotypic and genetic gains between crossbreeding strategies across the simulated scenarios suggest the potential for synthetic breeding to promote long-term genetic improvement in small-scale farms under diverse settings. Despite the current simulation referring to body weight and tick count incidence, many other productive and adaptive traits in African small-scale farms present similar genetic associations, implying comparable outcomes from same crossbreeding strategies for other trait combinations. For instance, moderate heritabilities are often reported for milk traits in African or tropical regions [78–80]. Diseases and functional traits (e.g., fertility) are low heritable [63, 81–83], as considered in the present study. Moreover, the range of considered genetic correlations between adaptive and productive traits (− 0.4 to 0.4) comprise a large number of estimates reported in the African or tropical context, e.g., [26, 82, 84, 85]. In addition, the tested genetic correlations between local and exotic environment offers a comprehensive overview for different scenarios of GxE effects. Nevertheless, the large diversity of cattle production systems and breeds in Africa request further studies to consider other traits or populations with specific genetic and demographic parameters, or to assess additional crossbreeding strategies [12]. The simulation pipeline developed in the present study will be of great help in this regard.

There are several limitations in this study, including use of simulation, unknown genomic and quantitative parameters, and assumptions made about organisation of crossbreeding strategies. Repeated failure of crossbreeding in smallholder settings indicates that existing approaches are not sustainable, which calls for a systematic study to evaluate alternative approaches in a stochastic simulation framework. While the results of such simulations are only indicative, the large differences between the compared scenarios in this study indicate a sustainable crossbreeding strategy for smallholder settings. All simulations depend on the implied assumptions, operationally encoded with various parameters. While we have good knowledge of many genomic and quantitative genetic parameters, we still lack nuanced information about the difference between demographies of *Bos Taurus* and *Bos Indicus* cattle sub-species [24] as well as the architecture of dominance variation. We have tuned our simulation such that the resulting outcomes matched published estimates of genomic and quantitative genetic parameters, but with scant literature on this topic, we expect a need for further more nuanced studies in the future as more information becomes available.

Moreover, we considered only one productive trait and one adaptive trait in this study. This simplification facilitates the comparisons of different scenarios including alterations of genetic correlations between traits, GxE effects, and selection indexes. However, it does not reflect all possible practical breeding approaches, as farmers usually consider more traits in their breeding objectives [26, 86, 87]. However, our results could also be seen in the context of two trait complexes. Simulation of more traits is technically possible, but limited by current knowledge on quantitative genetic parameters for many traits in the considered settings. In addition to additive and dominance effects, the simulation of epistatic effects would enable to analyse possible recombination losses in crossbreeds [88, 89]. Although a few studies described epistasis in beef and dairy cattle, accurate estimations of genetic variances due to epistatic effects are still limited [27, 90–93], as is the architecture of these effects. Similarly, while GxE effects in African context with smallholders are well known, there is a lack of literature on the architecture of these effects. In the light of these limitations, we have omitted epistasis in this study and simulated GxE under simplified assumptions. However, on the basis of more accurate information in this regard, advanced approaches to simulating these effects with scale and cross-over components, will be possible [94]. There is great potential in studying and leveraging these aspects for African smallholder production sector as indicated by other studies in highly diverse environments, e.g. [95, 96].

The constant need for local breeding females is a significant challenge in implementing F1 crossing in African smallholder farms [1]. To address this challenge, we assumed in this study that each smallholder farm kept a pure line of local animals while also producing F1. Although this approach provides indications on potential performances of F1 in smallholder farms, it is not feasible in practical settings. Indeed, smallholder farmers are unable to create and maintain separate breeding herds due to the limited resources and small scale of their farms. Moreover, we simulated rotational crosses on the smallholder farms by mating crossbred cows with local bulls at even generations, and with exotic bulls at odd generations. This approach facilitates a systematic introduction of exotic genes while preserving adaptive features from the local breed. However, mixing the crossbreeding pattern, for example by adjusting the frequency of use of exotic bulls, may reduce the observed fluctuations in phenotypic values and increase the adaptive performance of the crossbred animals. In addition, the incorporation of genomic data and advanced statistical models could lead to a better understanding of gene flow and trait expression in the crossbred populations. Finally, we assumed that a government farm would be available to preserve the local breed for its adaptive traits. Genomic technologies can significantly enhance the development of such nucleus farms in African countries. For instance, high-throughput genotyping and genome-wide association studies can accelerate the identification of causal variants and support effective breeding decisions [97–99]. The use of genomic information in breeding programs for local and crossbred cattle in Africa can enhance accuracy and efficiency of animal selection for improved productivity and adaptability [12, 34, 100]. These innovations will be of particular benefit to Africa, increasing food security and resilience in the continent.

## Conclusion

The current study provides insights into the possible success of different crossbreeding strategies for smallholder cattle production systems, like in many African countries, to improve both productive and adaptive traits. We demonstrate that long-term genetic improvement in smallholder cattle farms can be achieved through a proper design of synthetic breeding with effective management of inbreeding. Synthetic animals maximize responses to selection and long-term genetic gains because of their higher additive genetic variances compared to pure breeds. Also, the exchanged-village bull (EVB) synthetic scheme proposed in this study illustrates that extensive exchanges of genetic materials controls inbreeding, maintains heterosis in advanced generations, and promotes more genetic improvement in synthetic animals. Moreover, synthetic breeding offers the opportunity of combining various traits of interest in selection indexes for optimal genetic improvement of both adaptive and productive traits while minimising antagonist responses to selection. The presence of GxE effects between local and exotic environments implies an increased variation and possibly reduced mean phenotypic values of F1 or rotational crosses but does not affect genetic gain in F2 and subsequent generations of synthetic animals. Furthermore, when the two traits are negatively correlated, GxE effects enhance the adaptability of crossbred animals, but decrease their productivity.

## Supplementary Information

Additional file 1: Table S1. Heterosis for body weight in F1 crosses simulated in smallholder farms. Table S2. Heterosis for tick count incidence in F1 crosses simulated in smallholder farms.

Additional file 2: Figure S1. Phenotypic means for body weight and tick count incidence over the first 20 generations in the local and exotic breed.

Additional file 3: Table S3. Phenotypic and genetic parameters for simulated local and exotic breeds with regard to genetic correlation between traits (${r}_{g}$) and between local and exotic environement (${r}_{g\times e}$).

Additional file 4: Figure S2. Impact of the genetic correlation (${r}_{g}$) between body weight and tick count incidence on their phenotypic mean over 20 generations in different crossbreeds and local cattle populations simulated on smallholder farms. Figure S3. Impact of genetic correlation between local and exotic environment (${r}_{g\times e}$) on the phenotypic mean for body weight and tick count incidence over 20 generations in different crossbreeds and local cattle populations simulated on smallholder farms. Figure S4. Genetic-by-environment interactions significantly affect phenotypic gain in F1 and rotational crosses but not in synthetic crosses.

## Data Availability

All the data generated and supporting the findings in this simulation study are openly available in the JLUdata repository of the University of Giessen. The scripts for simulation are available at GitHub (https://github.com/SeyiV/Simulation_Crossbreeding).
