# Supplementary material for: Evaluation of crossbreeding strategies for improved adaptation and productivity in African smallholder cattle farms
Source: Genet Sel Evol. 2025 Feb 20;57:6. doi: 10.1186/s12711-025-00952-8 (PMC11844127; doi:10.1186/s12711-025-00952-8)
Supplement: Supplementary file 1 — Additional file 1: Table S1. Heterosis for body weight in F1 crosses simulated in smallholder farms. Table S2. Heterosis for tick count incidence in F1 crosses simulated in smallholder farms. [file 12711_2025_952_MOESM1_ESM.pdf]

Table S1. Heterosis for body weight in F1 crosses simulated in smallholder farms

r_g [1]	$r_{g\times e}$ [2]	Phenotypic value (Kg)				Heterosis	
		Hybrid value (BW_L)	Cows (BW_L)	Exotic Bulls (BW_E)	Midparent value	Value	Percentage (%)
0	0.4	511.59	323.93	627.71	475.82	35.77	7.53
0	0.6	530.16	323.82	627.94	475.88	54.28	11.43
0	0.8	550.41	324.30	628.54	476.42	73.99	15.55
0.4	0.4	521.56	354.61	627.92	491.27	30.29	6.20
0.4	0.6	544.65	352.52	627.12	489.82	54.82	11.21
0.4	0.8	564.37	351.17	628.33	489.75	74.62	15.25
-0.4	0.4	496.00	295.99	626.81	461.40	34.60	7.52
-0.4	0.6	518.06	298.08	626.72	462.40	55.66	12.05
-0.4	0.8	538.05	294.66	626.79	460.72	77.33	16.81

[1] r_g= genetic correlation between body weight and tick count incidence; $r_{g\times e}$ = genetic correlation between local and exotic environment; BW_L = body weight in the local environment, BW_E = body weight in the exotic environment.

Table S2. Heterosis for tick count incidence in F1 crosses simulated in smallholder farms

r_g[1]	$r_{g\times e}$[2]	Phenotypic value (-log10 [tick count])				Heterosis	
		Hybrid value (TC_L)	Cows (TC_L)	Exotic Bulls (TC_E)	Midparent value	Value	Percentage (%)
0	0.4	-0.25	-0.33	-1.52	-0.92	0.68	-73.24
0	0.6	-0.26	-0.33	-1.52	-0.92	0.67	-72.33
0	0.8	-0.25	-0.33	-1.53	-0.93	0.68	-72.58
0.4	0.4	-0.17	-0.32	-0.93	-0.63	0.46	-73.96
0.4	0.6	-0.12	-0.32	-0.95	-0.63	0.52	-81.50
0.4	0.8	-0.06	-0.33	-0.94	-0.64	0.58	-91.56
-0.4	0.4	-0.41	-0.34	-2.12	-1.23	0.82	-66.64
-0.4	0.6	-0.45	-0.34	-2.14	-1.24	0.79	-63.62
-0.4	0.8	-0.51	-0.33	-2.13	-1.23	0.72	-58.65

[1] r_g = genetic correlation between body weight and tick count incidence; $r_{g\times e}$ = genetic correlation between local and exotic environment; TC_L = tick count incidence in the local environment, TC_E = tick count incidence in the exotic environment
