# Supplementary material for: Evaluation of crossbreeding strategies for improved adaptation and productivity in African smallholder cattle farms
Source: Genet Sel Evol. 2025 Feb 20;57:6. doi: 10.1186/s12711-025-00952-8 (PMC11844127; doi:10.1186/s12711-025-00952-8)
Supplement: Supplementary file 2 — Additional file 2: Figure S1. Phenotypic means for body weight and tick count incidence over the first 20 generations in the local and exotic breed. [file 12711_2025_952_MOESM2_ESM.pdf]

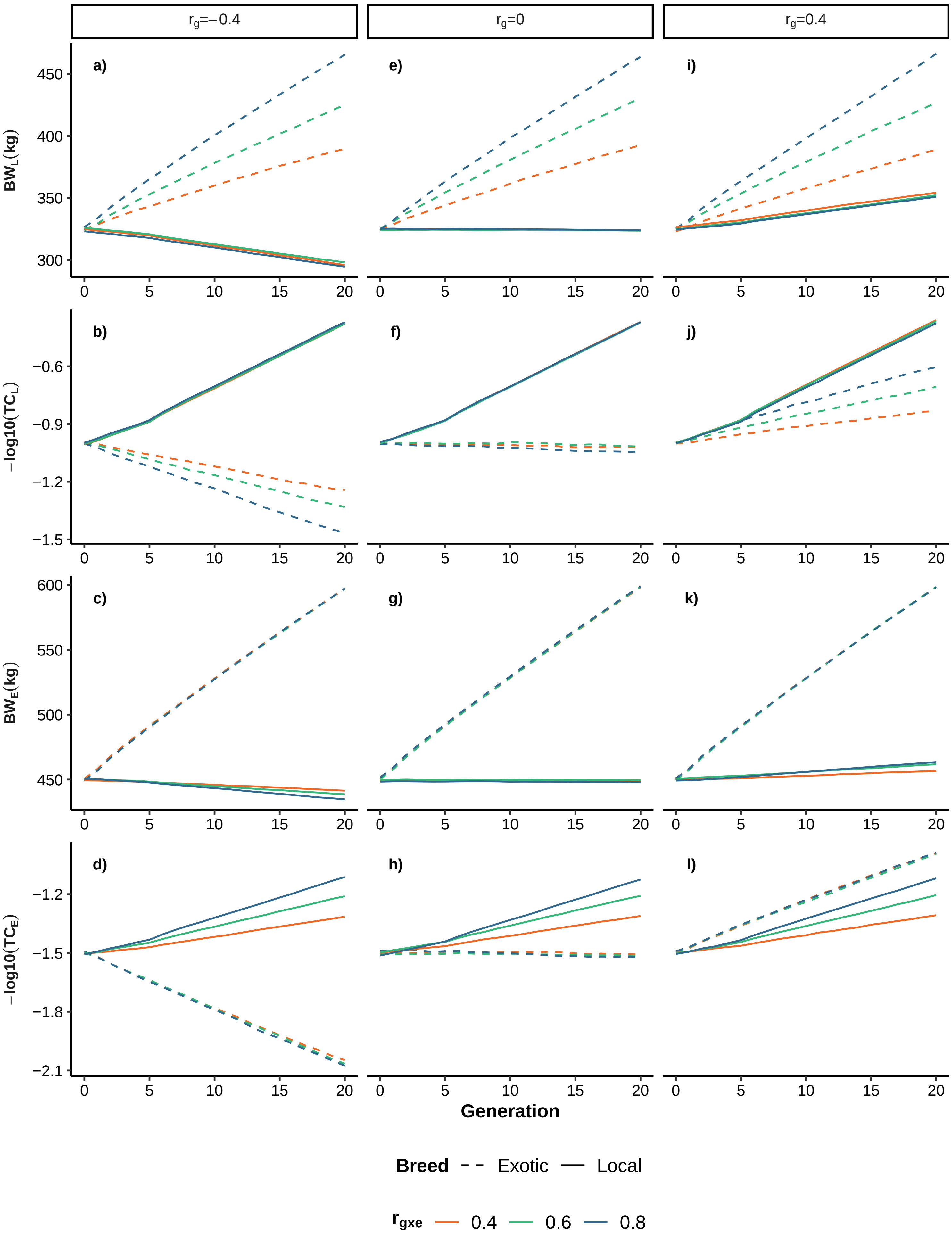

Figure S1: Phenotypic means for body weight and tick count incidence over the first 20 generations in the local and exotic breed

BW_L = body weight in the local environment; TC_L = tick count incidence in the local environment

BW_E = Body weight in the exotic environment; TC_E = tick count incidence in the exotic environment

r_g = genetic correlation between body weight and tick count incidence; r_{gxe} = genetic correlation between local and exotic environment
