# Supplementary material for: Evaluation of crossbreeding strategies for improved adaptation and productivity in African smallholder cattle farms
Source: Genet Sel Evol. 2025 Feb 20;57:6. doi: 10.1186/s12711-025-00952-8 (PMC11844127; doi:10.1186/s12711-025-00952-8)
Supplement: Supplementary file 4 — Additional file 4: Figure S2. Impact of the genetic correlation (${r}_{g}$rg) between body weight and tick count incidence on their phenotypic mean over 20 generations in different crossbreeds and local cattle populations simulated on smallholder farms. Figure S3. Impact of genetic correlation between local and exotic environment (${r}_{g\times e}$rg×e) on the phenotypic mean for body weight and tick count incidence over 20 generations in different crossbreeds and local cattle populations simulated on smallholder farms. Figure S4. Genetic-by-environment interactions significantly affect phenotypic gain in F1 and rotational crosses but not in synthetic crosses. [file 12711_2025_952_MOESM4_ESM.pdf]

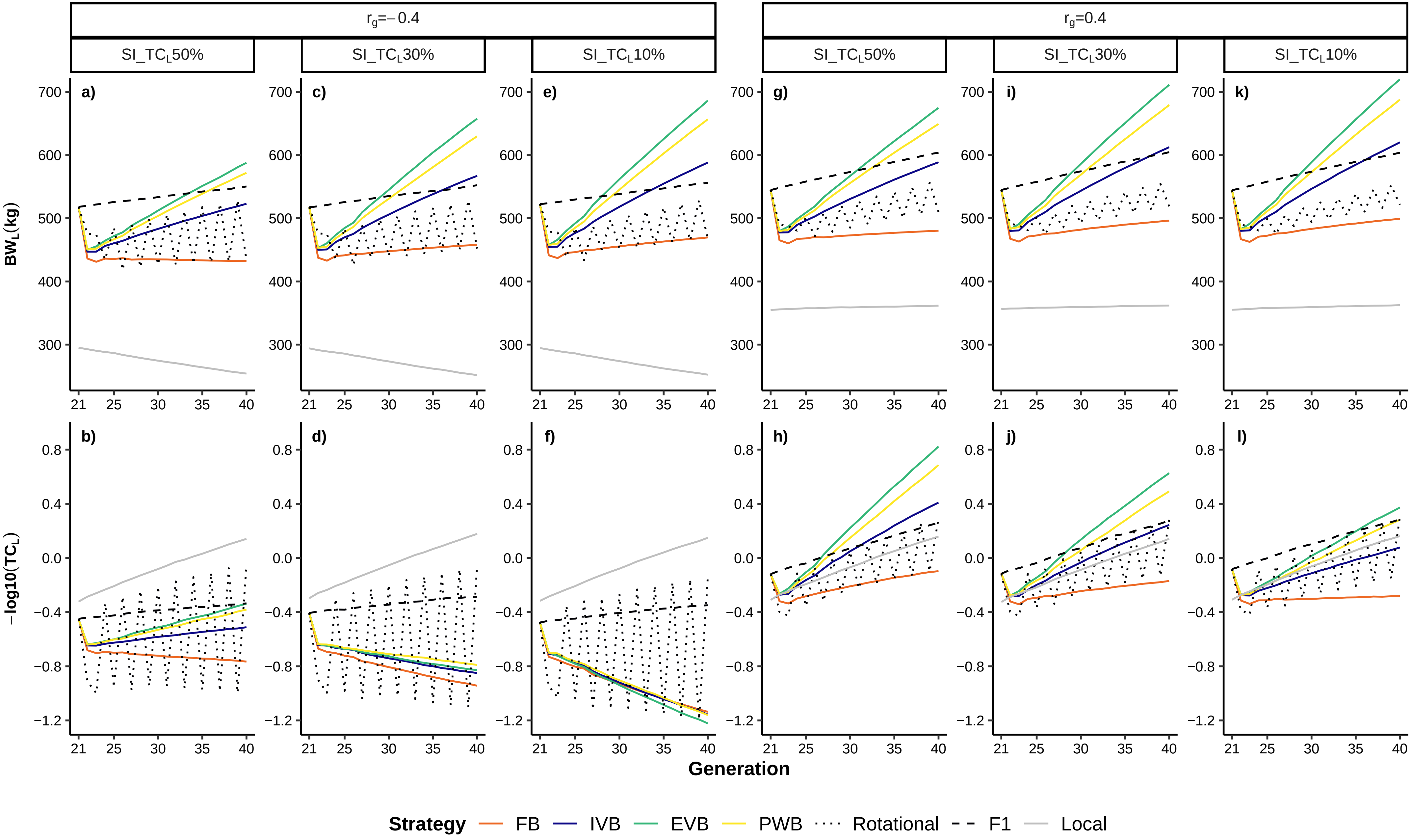

Figure S2. Impact of the genetic correlation between body weight and tick count incidence on their phenotypic mean over 20 generations in different crossbreeds and local cattle populations simulated on smallholder farms.

BW_L = body weight in the local environment; TC_L = tick count incidence in the local environment; genetic correlation between local and exotic environment(r_{gxe})=0.6;

FB = Farm bull composite scheme, IVB = Intra−village bull composite scheme, EVB = Extra−village bull composite scheme, PWB = Population−wide bull composite scheme.

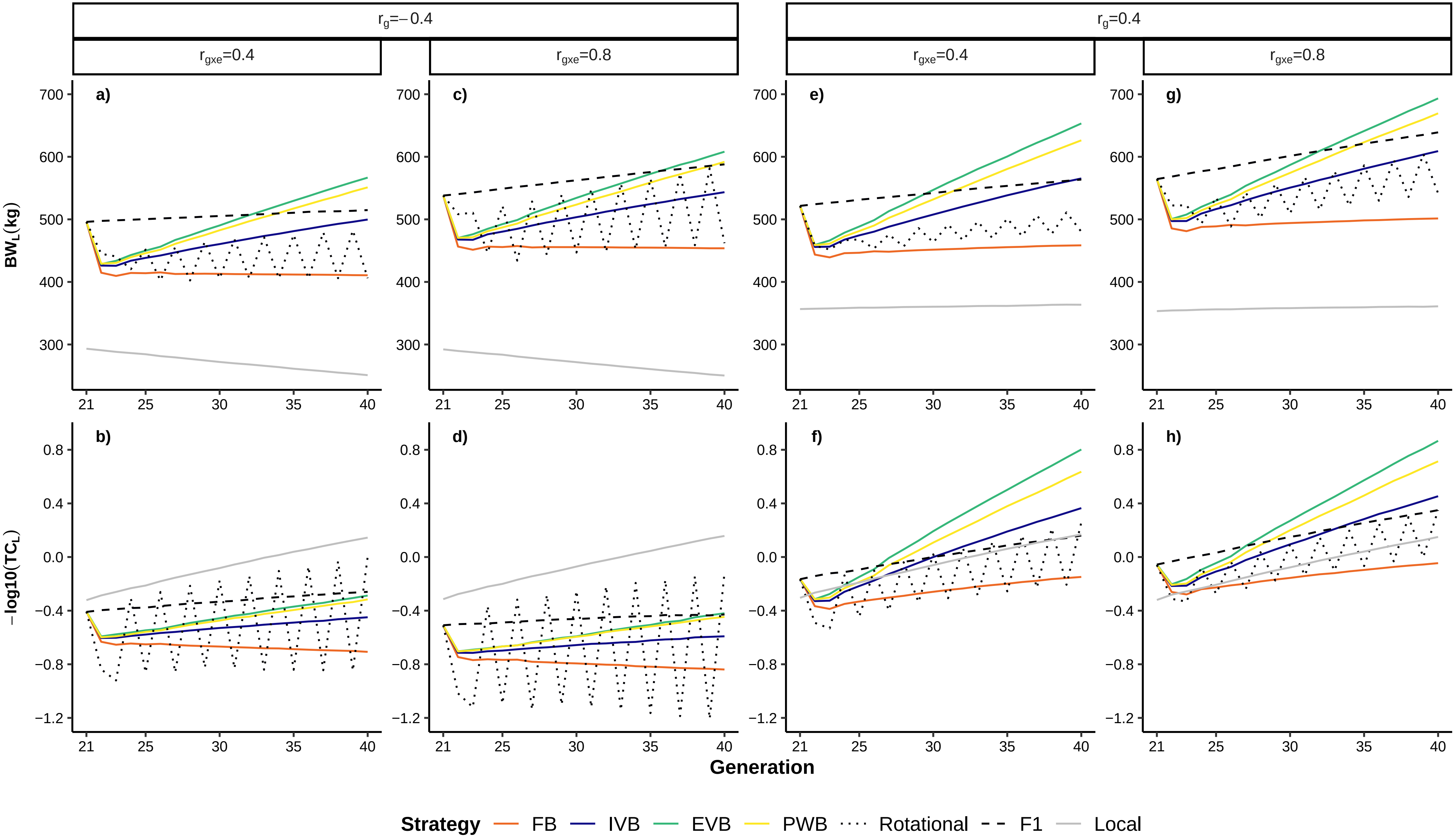

Figure S3. Impact of genetic correlation between local and exotic environment on the phenotypic mean for body weight and tick count incidence over 20 generations in different crossbreeds and local cattle populations simulated on smallholder farms.

BW_L = body weight in the local environment; TC_L = tick count incidence in the local environment;
r_g = genetic correlation between body weight and tick count incidence; the selection index SI TC_L50 % was applied (see Table 2 for more details);
FB = Farm bull composite scheme, IVB = Intra−village bull composite scheme, EVB = Extra−village bull composite scheme, PWB = Population−wide bull composite scheme.

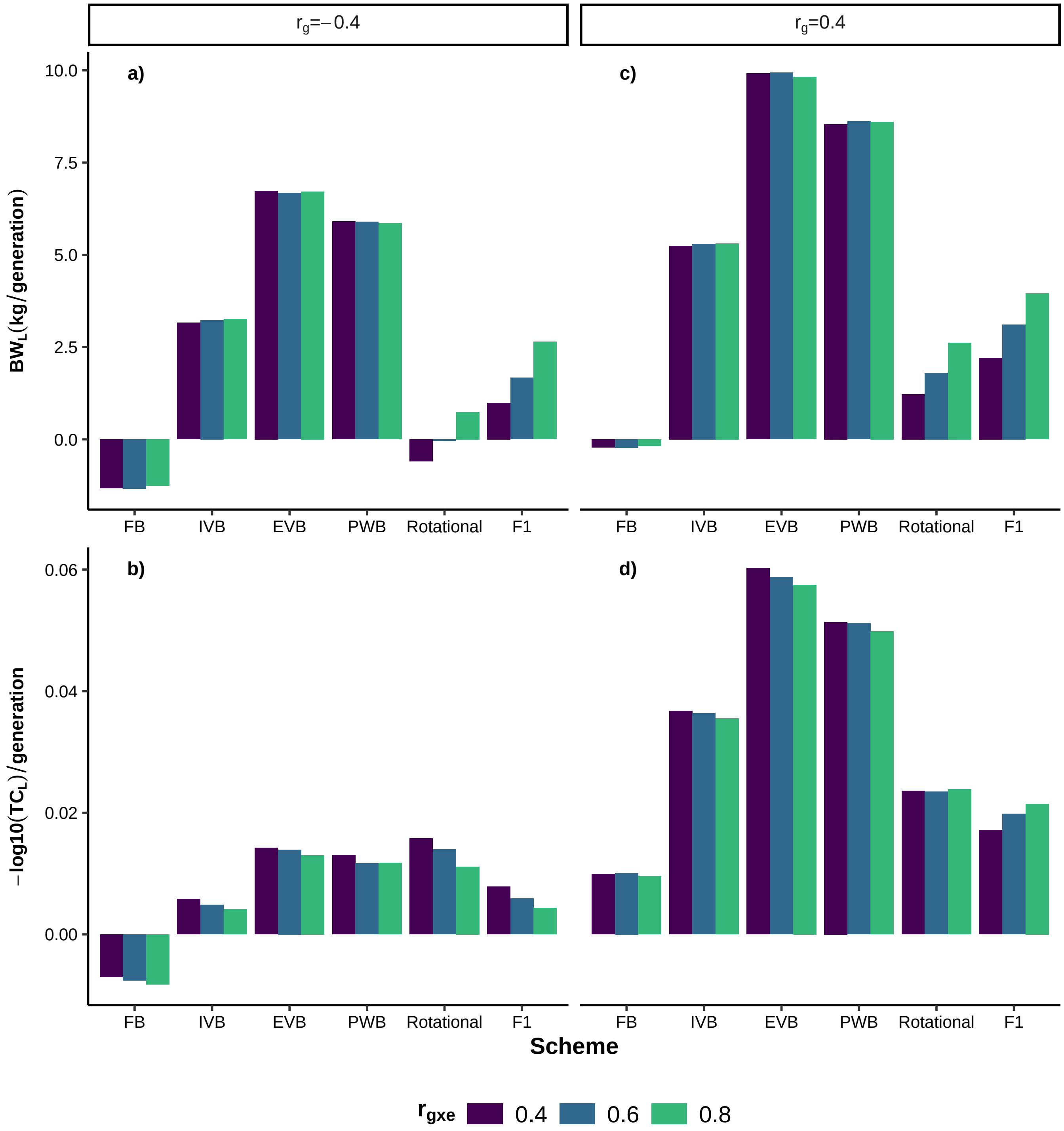

Figure S4. Genetic−by−environment interactions significantly affect phenotypic gain in F1 and rotational crosses but not in composite crosses.

BW_L = body weight in the local environment; TC_L = tick count incidence in the local environment;
r_g = genetic correlation between body weight and tick count incidence;
the selection index SI TC_L50 % was applied (see Table 2 for more details);
FB = Farm bull composite scheme, IVB = Intra−village bull composite scheme);
EVB = Extra−village bull composite scheme, PWB = Population−wide bull composite scheme.
